# Reconciling advantages and difficulties: knowledge and perceptions of event-driven PrEP among young people

**DOI:** 10.11606/s1518-8787.2024058005729

**Published:** 2024-10-11

**Authors:** Luiz Fabio Alves de Deus, Dulce Ferraz, Lorruan Alves dos Santos, Alexandre Grangeiro, Inês Dourado, Marcia Thereza Couto, Ramiro Fernandez Unsain, Eliana Miura Zucchi

**Affiliations:** I Associação Brasileira Interdisciplinar de AIDS (ABIA). Rio de Janeiro, Brasil Associação Brasileira Interdisciplinar de AIDS (ABIA) Rio de Janeiro Brasil; II Grupo Interdisciplinar de Estudos sobre HIV e Direitos Sexuais e Reprodutivos. Universidade Católica de Santos. Santos, Brasil Universidade Católica de Santos Grupo Interdisciplinar de Estudos sobre HIV e Direitos Sexuais e Reprodutivos Santos Brasil; III Escola de Governo Fiocruz Brasília. Fundação Oswaldo Cruz. Brasília, DF, Brasil Escola de Governo Fiocruz Brasília Fundação Oswaldo Cruz Brasília DF Brasil; IV UMR1296 «Radiations, Défense, Santé, Environnement». Université Lumière Lyon 2. Lyon, France Université Lumière Lyon 2 UMR1296 «Radiations, Défense, Santé, Environnement» Lyon France; V Grupo de estudo e pesquisa em Saúde, Interseccionalidade e Marcadores Sociais da Diferença. Universidade de São Paulo. São Paulo, SP, Brasil Universidade de São Paulo Grupo de estudo e pesquisa em Saúde, Interseccionalidade e Marcadores Sociais da Diferença São Paulo SP Brasil; VI Faculdade de Medicina. Universidade de São Paulo. São Paulo, SP, Brasil Universidade de São Paulo Faculdade de Medicina São Paulo SP Brasil; VII Universidade Federal da Bahia. Instituto de Saúde Coletiva. Salvador, BA, Brasil Universidade Federal da Bahia Instituto de Saúde Coletiva Salvador BA Brasil; VIII Universidade de São Paulo. Faculdade de Medicina. Departamento de Medicina Preventiva. São Paulo, SP, Brasil Universidade de São Paulo Faculdade de Medicina Departamento de Medicina Preventiva São Paulo SP Brasil; IX Universidade de São Paulo. Faculdade de Medicina. Programa de Pós-graduação em Medicina Preventiva. São Paulo, SP, Brasil Universidade de São Paulo Faculdade de Medicina Programa de Pós-graduação em Medicina Preventiva São Paulo SP Brasil; X Universidade Católica de Santos. Programa de Pós-Graduação em Saúde Coletiva. Santos, SP, Brasil Universidade Católica de Santos Programa de Pós-Graduação em Saúde Coletiva Santos SP Brasil

**Keywords:** Pre-exposure prophylaxis, HIV, Sexual and gender minorities, Adolescents, Qualitative research

## Abstract

**OBJECTIVE:**

This study aims to analyze the knowledge about the HIV event-driven pre-exposure prophylaxis (event-driven PrEP) scheme and the perception of its potential use among young men who have sex with men (MSM), *travestis*, and transgender women (TrTW) who were followed up in the cohort.

**METHODS:**

This qualitative study included 50 interviews with participants from the municipalities of Salvador and São Paulo, aged 15 to 19 years, who made daily use of PrEP or other preventive methods. They were addressed by different demand creation strategies. The in-depth interviews covered topics such as sexual practices, event-driven PrEP knowledge, acceptability, and motivations for its use. A two-stage thematic analysis was carried out on Nvivo, version 12.

**RESULTS:**

Most participants were unaware of event-driven PrEP, and many questioned its effectiveness and safety when receiving information about it. However, on learning about the program, many young people saw advantages, such as not having to take daily medication and the possibility of using it only at times of greater risk. Participants also found barriers to using event-driven PrEP, such as the unpredictability of sexual relations and the difficulty in administering dosages in this modality.

**CONCLUSION:**

Limited knowledge and experiences with daily oral PrEP influenced interest in event-driven PrEP, which highlights the need for information strategies that enable young MSM and TrTW to read about event-driven PrEP. Young people valued the autonomy and management of preventive methods provides by this new modality, which is more in line with the dynamics of their sexual lives, but they face challenges in managing event-driven PrEP.

## INTRODUCTION

Expanding access to HIV Pre-Exposure Prophylaxis (PrEP) in Brazil is a crucial element in the response to the HIV epidemic among the most affected groups, such as men who have sex with men (MSM), *travestis* and transgender women (TrTW), sex workers, and serodifferent partners. In addition, special attention must be devoted to the population of young men aged 15 to 24, for whom the epidemic has been growing^1^.

Although PrEP coverage has increased among the adult population in several countries^2^, adolescent and young MSM and TrTW face significant challenges in accessing, adhering to and retaining prophylaxis, due to legal, economic and social restrictions that create barriers along the PrEP care continuum^3^^), (^^4^. The difficulty of access and adherence to PrEP among MSM and TrTW, especially those under 18 years old, stems from an interrelated range of individual, social and programmatic factors, requiring a comprehensive and integrated health approach^5^^), (^^6^. Evidence also indicates that low levels of perceived risk of HIV infection and knowledge about PrEP directly affect interest in prophylaxis among MSM and TrTW individuals^7^. High discontinuation rates of PrEP raise questions on the effectiveness of this method in changing the trajectory of the HIV epidemic among these groups^8^. A study conducted with adolescents and young MSM in Chicago identified barriers to accessing health services, difficulty in attending follow-up appointments and low perception of HIV risk as reasons for discontinuing prophylaxis^9^. Thus, the barriers to adherence to daily PrEP faced by MSM and TrTW, combined with the specificities of these groups, highlight the need to offer alternatives.

In this context, the PrEP event-driven modality (event-driven PrEP) was recently incorporated into HIV prevention policy in Brazil, being offered as an alternative to daily PrEP for specific groups, including MSM and TrTW, as long as they are not taking estradiol-based hormones^10^. This PrEP scheme consists of taking two Tenofovir/Emtricitabine (TDF/FTC) tablets between 2 and 24 hours before risky sex, followed by two more doses, one 24 and the other 48 hours after the first dose^11^. When used correctly, event-driven PrEP shows similar efficacy to daily PrEP^12^ in adult MSM and TrTW, and is recommended for individuals with sporadic and predictable sexual practices^11^^), (^^13^. However, there are no specific studies on the effectiveness and safety of using event-driven PrEP in MSM and TrTW under the age of 18^14^.

Increasing knowledge and acceptance of the different PrEP modalities among young and adolescent MSM and TrTW people is important for promoting the right of these groups to prevention. Currently, evidence shows low rates of knowledge about the event-driven PrEP modality in these groups, as well as doubts on its efficacy and effectiveness, and challenges related to predicting sexual relations and taking pills as recommended^15^. Despite this, when informed on the different PrEP schemes, rates of interest and acceptability of event-driven PrEP are high among young people^16^.

In order to understand how adolescents and young people respond to information about event-driven PrEP and its potential acceptance, it is essential to consider the construction of their autonomy, the dynamics of sexual encounters, the repercussions of sexual orientation and gender identity in different life contexts, especially in the family. It is also important to examine how these experiences contribute to the construction of perceptions about different HIV prevention strategies.

We understand perception as the individual appropriation and interpretation of a phenomenon that influences health behaviors and practices^17^. Thus, this knowledge can help identifying the intention to use and potential obstacles to access to prophylaxis, contributing to plan strategic actions to increase access, retention, and adherence to PrEP in these groups. To move forward in this direction, this study aimed at analyzing knowledge about the event-driven PrEP scheme and the perception of its potential use among young MSM and TrTW being followed up in the cohort PrEP1519.

## METHODS

This study is part of a qualitative investigation into access to services and linkage to care for HIV and sexually transmitted infections (STIs) in the context of community interventions to offer testing and PrEP to young people and adolescents participating in the PrEP1519 study in São Paulo and Salvador^18^.

### The study PrEP1519

This study PrEP1519 begun in 2019 and is the first cohort in Latin America to show the effectiveness of daily oral PrEP among MSM and TrTW adolescents aged 15 to 19 living in three Brazilian capitals: Salvador, São Paulo and Belo Horizonte^19^. The active recruitment of the target population for PrEP enrollment used different demand creation strategies. Various virtual platforms were used (Facebook, WhatsApp, Instagram, Twitter, YouTube, and Spotify), and peer educators interacted with potential participants on dating apps (Grindr, Hornet, Tinder and Badoo). Face-to-face initiatives involved peer education led by adolescents and young adults, who approached other adolescents in places of sociability (bars, parks, beaches, streets, etc.), as well as referrals from health service professionals specializing in HIV.

The data was collected from 50 in-depth interviews with participants from Salvador and São Paulo, between July 2019 and September 2020. We sought to diversify in relation to: (i) gender identities (cis men, *travestis* and transgender women); (ii) sociodemographic conditions; (iii) experiences of adherence to PrEP; (iv) use of other preventive methods; and (v) those who entered the study through different demand creation strategies. Health professionals and peer educators helped to identify potential participants according to these criteria, as well as facilitating the invitation to take part in the qualitative research.

The interviews were conducted using scripts that covered topics such as sexual practices, use of prevention methods, acceptance of other PrEP modalities (event-driven PrEP, injectable and subcutaneous), quality of life and future plans. We asked PrEP users on adherence to treatment and experiences related to use in their daily lives, as well as interaction with health professionals. Participants who chose not to use PrEP at the time of inclusion in the cohort were asked why they refused and their choice of other prevention methods. During the interviews, participants were also asked if they knew any other PrEP modalities besides daily use, followed by an explanation of event-driven PrEP.

Trained and experienced interviewers carried out the interviews, which were mostly conducted face-to-face. However, due to the restrictions imposed by the covid-19 pandemic, some were carried out by video call. They lasted between 50 and 90 minutes. All participants gave their consent to take part and record the interviews, which were then transcribed, reviewed, and anonymized.

Data analysis was conducted in two distinct and complementary phases. In the first phase, codes were developed with the central themes addressed in the interviews, using a deductive approach based on the topics in the scripts. The narratives were then coded and stratified into the appropriate codes. To do this, the participants were identified using a cross-extraction method, allowing for contextual and individual analysis of the narratives, as well as comparing perceptions of event-driven PrEP among the groups studied.

The data was coded using NVivo software, version 12. Based on the coding matrix, the second phase of data exploration was aligned with the objective of the study, using the principles of thematic analysis^20^. Three structuring themes emerged: (1) the (lack of) knowledge about the event-driven PrEP scheme; (2) the advantages and motivations for using event-driven PrEP; and (3) the potential difficulties perceived in using the scheme. It should be noted that, due to the innovative nature of event-driven PrEP and the other possibilities for HIV prevention, the narratives overlap and the themes identified are not mutually exclusive.

The participants were quoted in the results section through their speeches, identified by code name, age, race/skin color, use of PrEP or other prevention methods, and PrEP1519 study site.

The study PrEP1519 was approved by the Research Ethics Committees of the Universidade de São Paulo (protocol 70798017.3.0000.0065) and the Univerdidade Federal of Bahia (protocol 01691718.1.0000.5030). Participants over the age of 18 signed an informed consent form. For adolescents aged between 15 and 17, different procedures were adopted: in Salvador, they signed the informed assent form, while their parents or guardians signed the informed consent form; in São Paulo, with judicial authorization, the adolescents were able to consent without the obligatory consent of their parents or guardians.

## RESULTS

Out of 50 interviewees, 29 were from Salvador and 21 from São Paulo. At the interview, 36 were using daily PrEP and 14 were using other preventive methods. The majority were cisgender men (n=42), Black or Mixed-race (n=40), aged between 18 and 20 (n=43), almost half were in higher education (n=23) and the majority identified themselves as gay (n=33) (Table 1).

**Table 1:** Sociodemographic profile of participants, use of daily oral HIV pre-exposure prophylaxis (PrEP) and knowledge about event-driven PrEP. Salvador, BA, and São Paulo, SP, Brazil, 2019-2020.

Characteristics	Salvador (N=29)	São Paulo (N=21)
Gender identity		
Cisgender man	19 (49%)	20 (51%)
Transgender woman	06 (100%)	0 (0%)
*Travesti*	0 (0%)	1 (100%)
Non-binary	04 (100%)	0 (0%)
Age group (years)		
15-17	3 (43%)	04 (57%)
18-20	26 (60%)	17 (40%)
Race/skin color		
White	05 (50%)	05 (50%)
Black	18 (62%)	11 (38%)
Mixed-race	06 (55%)	05 (45%)
Education		
Incomplete secondary education	06 (55%)	05 (45%)
Completed secondary education	08 (62%)	05 (38%)
Incomplete higher education	13 (54%)	11 (46%)
No information	02 (100%)	0 (0%)
Sexual orientation		
Heterosexual	4 (80%)	1 (20%)
Bisexual	6 (60%)	4 (40%)
Gay	18 (55%)	15 (45%)
Pansexual	1 (50%)	1 (50%)
Taking PrEP		
Yes	19 (53%)	17 (47%)
No	10 (71%)	04 (29%)
Previous knowledge about PrEP event-driven		
Some information	02 (50%)	02 (50%)
Completely unknown	27 (59%)	19 (41%)

### Knowledge (or lack thereof) of the event-driven PrEP scheme

Almost all participants were unaware of event-driven PrEP (46/50). Some from São Paulo confused event-driven PrEP with the injectable form of PrEP: “I think the one you apply every 3 months?” (Renato, 19, Black, No PrEP, São Paulo); “Is this event-driven one the one you talked about applying to the buttocks?” (Girassol, 20, White, discontinuing PrEP, São Paulo).

When they received information about event-driven PrEP, some participants (six) were surprised and had doubts about its effectiveness and safety:

I think this event-driven is a bit strange. [...] No... the right thing to do would be to be prevented, rather than take the drug two 2 hours before [...] (Romeu, 19, Black, non-user of PrEP, Salvador).

From the information I know, it protects with seven days in a row and not two days before and two days after intercourse. I don’t think [event-driven PrEP] protects (Bernardo, 17, White, PrEP user, São Paulo).

Doubts on the interaction between the drugs used in the event-driven PrEP scheme and those prescribed for hormone therapy were pointed out by a transgender woman, as well as concerns about side effects that emerged in the narratives of both transgender women and cis men: “I’m very afraid of [damaging] my kidneys [...]. Because of the hormone and PrEP too, right?” (Vera, 20, Black, non-user of PrEP, Salvador); “Some side effect [of event-driven PrEP] could be different, right? The side effect could be bigger [...]” (Kleber, 20, White, daily PrEP user, Salvador).

Few interviewees (four) had had previous contact with information about event-driven PrEP. The number of pills, the interval between the first intake and sexual intercourse, the number of doses needed after sexual intercourse and the time interval for subsequent use were unknown:

I think I’ve heard of just one [PrEP scheme use]. I think it was two [pills] yes, one no. Something like that, you know? (Jackson, 17, Black, PrEP user, São Paulo).

These days I read about taking PrEP three days before or a few hours before, I don’t remember. It was a combination taken a few moments before sex and after sex only, it wasn’t something continuous. I don’t remember the exact time or order, but I know it was something only at the moment of sex and a little bit after sex (Conrado, 19, White, non-user of PrEP, Salvador).

Among those who said they knew about event-driven PrEP, only one showed sufficient knowledge to decide to use the scheme. For him, daily use represented a threat to exposing his sexual orientation to his family. Combining the perception of the risk of acquiring HIV and information from his social network, the young man took interest in the occasional use of PrEP in the follow-up appointment for daily oral PrEP: “I negotiated with him [the doctor]. Then he explained the event-driven PrEP to me” (Hugo, 20, Mixed-race, PrEP user, São Paulo).

### Perceived advantages and motivations for using the event-driven scheme

When introduced to event-driven PrEP, participants mentioned perceived advantages of the scheme over daily PrEP. Using PrEP only when necessary and not having to take it every day were cited as potential advantages. The understanding of these advantages was based on the assessment that there are periods of infrequent sexual intercourse, allowing for planning. The sporadic nature of exposure to risk, combined with the tiredness of taking pills every day, led many interviewees to consider the event-driven PrEP scheme interesting, as seen in the following statements:

I liked it. I really liked it. I think I would adhere to it. [...] Even more so in the current situation. Because I’m not having much sex (Thiago, 18, Black, PrEP user, São Paulo).

Oh, I think it’s much cooler. Why? [...] It’s very complicated for me to take medication every day. I just don’t like it. (Guilherme, 20, Black, non-user of PrEP, São Paulo).

I think that in case I no longer want to use PrEP, taking a pill every day, event-driven PrEP already helps, right? (Fernando, 19, Mixed-race, PrEP user, Salvador)

In addition, the scheme was seen as beneficial for situations with a higher risk of HIV infection, such as sex parties, saunas, and dark-rooms. For some participants who did not use PrEP, especially those in a monogamous relationship, event-driven PrEP was beneficial because it is used only in the highest risk episodes:

And I think it’s much more chill, especially for me, I don’t like taking medication, and there must be other people who don’t like this continuous use either, but it would be a possibility if, one day, I want to have sex with someone outside the relationship, to prepare myself beforehand. Conrado, 19, White, non-user of PrEP, Salvador).

I would take it. Look, if I go to a [sex] party or have a relationship with someone I don’t know, so I’d use it. Then I’d know, I’d use it straight away. Or, if I’m on a dating site and I’ve arranged to go out with someone, so I’m going to go out, I’m going to take it (Danilo, 19, Black, PrEP user, Salvador).

In event-driven PrEP, lower chances of adverse effects were also perceived, due to the lower intake of pills compared to daily PrEP: “I think it is very viable, because you will be taking it every day, but it is not every day you will have sex. Then you end up taking it, and that can also create problems, right?” (Orlando, 18, Mixed-race, non-PrEP user, Salvador).

Furthermore, one participant noted a collective benefit associated with event-driven PrEP: reducing the number of consumed pills allow a more rational use of prophylaxis within the social group, enabling more people to use it.

“So, there’s no need for me to take so many pills from a person who could be using it, who could really need it, you know?” (Thiago, 18, Black, PrEP user, São Paulo).

### Potential difficulties for the use of event-driven PrEP

Participants mentioned potential difficulties and concerns about using event-driven PrEP. For some (21), the unpredictability of sexual relations was highlighted as an obstacle, as encounters occur suddenly, making it difficult to take the first dose up to two hours before exposure: “I think daily PrEP is more efficient. Because you don’t always know you’re going to have sex” (Theo, White, 18 years old, PrEP user, São Paulo); “I don’t think it would work for me, because I never predict [sexual relations], I never schedule it” (Paula, 18, White, PrEP user, Salvador).

Insecurity on protection in cases of exposure to risk were also mentioned. The number of pills and the instruction to take the first dose up to two hours before intercourse led to apprehension on reduced efficacy and possible adverse effects compared to daily PrEP. Some (18) have expressed concern that reducing the number of pills in event-driven PrEP compared to daily use will result in less protection against HIV:

Ah, I think it might work. But, from the information I have, it wouldn’t take it. As far as I know, it protects seven days in a row, not two days before and two days after intercourse. I don’t think it protects (Bernardo, 17, White, PrEP user, São Paulo).

There are times when you think it won’t happen. For example, the last time I had sex I didn’t think I would. So, there are things you don’t know about if you don’t take the pill regularly (Walter, 19, Black, PrEP user, Salvador).

I think this demand thing [is] a bit strange [...]. No, [...] the right thing to do would be to already be prevented, rather than take the drug 2 hours before and think it’s going to have a good effect (Romeu, 19, Black, non-user of PrEP, Salvador).

In addition, some participants (eight) felt unable to follow the recommendation to use PrEP:

That’s tough. Really tough. Why? Oh, I don’t know, [because] you take a risk, people forget stuff (Luiz, 20, Black, PrEP user, São Paulo).

I wouldn’t use event-driven PrEP because I have trouble in remembering things. To me, adapting to taking the medication was complicated, and I still have to write down when I take it (Roberto, 16, Black, daily PrEP user, Salvador).

Finally, although less mentioned, there was concern about making the use of event-driven PrEP compatible in contexts of high sexual activity, both with a steady and casual partner:

Look, if it is [...], there are four. If you have sex twice a week, then there will be eight, eight pills, so [...] (Carlos, 19 years old, Black, PrEP user, Salvador).

For me, event-driven wouldn’t be necessary, because I date, I have a steady relationship, you know, it’s commonplace, so “event-driven” would become daily, understand? (Luciano, 18 years old, Mixed-race, PrEP user, Salvador).

## DISCUSSION

Knowledge about event-driven PrEP was not part of the HIV prevention repertoire of young MSM and TrTW. The few participants who had some prior information about event-driven PrEP said that they use it when they have sex, but without complete knowledge of how to use the scheme properly. As this knowledge is still in its infancy, the expression “event-driven” was confused with injectable PrEP among some, and information on effectiveness and forms of use was confused with daily use. Research using different methodological approaches has shown that knowledge about event-driven PrEP is low even among daily PrEP users^21^^), (^^22^^), (^^23^. And among those who reported having some knowledge of the modality, when encouraged to provide information about dosages and the pre- and post-sexual intercourse scheme, few reported it correctly, as in other studies^21^^), (^^22^^), (^^23^^), (^^24^. This has even been one of the main problems leading to incorrect use of the scheme^25^.

In addition, among the TrTW, there was concern that the interaction between drugs in event-driven PrEP and substances used for hormone therapy could result in kidney damage, a similar concern identified in a US study with a group similar to the one in this study^26^. However, this group did not highlight concerns on the possibility of interaction between hormone use and the efficacy of the scheme, which could contraindicate the use of event-driven PrEP for these people^27^. These results reiterate other findings that highlight how MSM and TrTW are negatively affected in terms of access to information on preventive methods based on the use of antiretrovirals^23^. In a context of high HIV prevalence among MSM and TrTW, as presented in studies carried out in Brazil^28^, the lack of knowledge about event-driven PrEP reveals the need to develop and include information on the different PrEP schemes in combined HIV prevention actions that take into account the specificities and needs of the most vulnerable population subgroups. This could lead to an increase in PrEP coverage in groups most susceptible to the epidemic, reaching people with different sexual behaviors and different prevention needs.

Corroborating such aspects, even showing incipient knowledge, young MSM and TrTW were able to quickly assimilate information about the scheme event-driven and, when evaluating the characteristics of their sexual relationships, mediated or not by the experience of using daily PrEP, they identified advantages and intention to use event-driven PrEP. In general, the benefits recognized were linked to the perception of greater autonomy regarding decision-making on the PrEP scheme to be used. As there are different PrEP schemes available, and considering that in the field of HIV prevention the best scheme is the one that takes into account values, different expectations, needs and realities of individuals, user-centered decision-making is an important strategy for the advancement and dissemination of care in the context of HIV prevention^29^. In considering the event-driven PrEP modality advantageous, participants took into account their own ability to manage a complex network of elements that are fundamental to decision-making, such as the frequency and seasonality of sexual activity and the ability to predict and plan sexual encounters.

Thus, approaches allowing a dialogical process between health professionals and users in the decision-making process on the PrEP scheme seem to have a better chance of success^29^^), (^^30^. Research carried out mostly with MSM has identified that aspects related to the dynamics of sexual encounters, such as the ability to plan them, irregularity or decrease in their frequency^25^^), (^^30^^), (^^31^^), (^^32^^), (^^33^^), (^^34^^), (^^35^^), (^^36^^), (^^37^^), (^^38^, low perception of risk of acquiring HIV^25^^), (^^36^^), (^^39^. They also revealed concerns on excessive use of medication^21^^), (^^29^^), (^^30^^), (^^32^^), (^^33^ and fear or experience with side effects^23^^), (^^36^^), (^^39^ influence the initial choice of PrEP or the change from daily to event-driven use. In addition, they can influence an appropriate process for managing PrEP use, allowing daily and event-driven use to be interchanged according to the profile of sexual relations at any given time.

Furthermore, the recognition of the greater convenience, flexibility and practicality of the event-driven PrEP scheme, as opposed to the perception that daily PrEP, in certain circumstances may not be the best option due to the daily intake of pills, was also identified in other qualitative research^13^^), (^^32^. It permitted event-driven PrEP to emerge as an interesting alternative for individuals who are in monogamous relationships, but eventually get involved in sexual relations that escape the exclusivity agreement. It is important to highlight the concern on HIV infections occurring in monogamous relationships as these situations have also been observed in cases of PrEP users, who interrupt prophylaxis when starting monogamous relationships because they do not perceive themselves to be at risk^40^. Therefore, it is crucial that this aspect be addressed during counseling, in order to allow adequate decision-making on the safest preventive strategies for these situations.

Similar to other investigations^41^, the results of this study show that event-driven PrEP was also perceived as having potential collective benefit, as it resulted in more rationalized use and was capable of expanding the offer to more people, through better use of the pills available in the system of health. Indirectly, this perception of collective benefit also seems to indicate young people’s perception of the need for adaptations in contexts of limited prevention inputs.

In contrast to the perceived advantages of event-driven PrEP, considerations were made about potential difficulties in using the modality. First, the eventual use of event-driven PrEP appeared to be hindered by the unpredictability of sexual encounters, a barrier that seemed to be common when it comes to event-driven PrEP^33^^), (^^35^^), (^^37^. The difficulty of predicting and planning sexual relations may be mediated by the immediacy of sexual encounters. It is worth noting that the increase in the use of apps to find sexual partners has been pointed out as an advantage for planning the use of event-driven PrEP, as it marks the moment when use of the modality begins. On the other hand, it is important to consider that many adolescents and young adults live with parents and/or other family members who are unaware of their sexual desires and practices^42^. In this context, limited autonomy and independence, including financial independence, can contribute to the perception that certain opportunities to experience sexuality are unique, which can create a barrier to using the initial dosages of event-driven PrEP as recommended. The ability to manage the use of all the pills in the scheme emerged as a hindrance to the event-driven PrEP scheme, perceived as complex and prone to forgetfulness, in line with the findings of other investigations^33^^), (^^35^^), (^^38^. Thus, an important specificity of young people and adolescents consists of recognizing autonomy for care as a learning process that requires dealing with the complexity of decisions and interaction with the contexts of family and social relationships, as well as the organization of the care routine integrated with the dynamics of sexual encounters to manage the eventual use of PrEP.

Furthermore, there is a clear need for strategies to publicize and promote PrEP to focus on deconstructing mistaken beliefs on its efficacy and effectiveness, such as those identified in this and other studies^32^. A study of Australian gay and bisexual men found that the belief in efficacy was associated with prior knowledge of the method^24^. Among our participants, doubts that emerged on the efficacy of event-driven PrEP seem to be linked to the novelty of the information on the method, the experience of use and greater exposure to information about daily PrEP, such as the need to use the drug for seven days to obtain the necessary concentration in the body and ensure adequate protection. Hence, the recommendation to make the daily and event-driven modality available in a single oral scheme seems appropriate, unifying the procedures for initiation (a loading dose of two tablets), maintenance (one tablet per day of intercourse) and completion (two tablets after the last potential exposure)^10^.

## CONCLUSIONS

Low knowledge and perceptions based on experience with daily PrEP influenced interest in and decision about event-driven PrEP, which highlights the need to improve information strategies and the provision of this scheme in combined prevention strategies for young MSM and TrTW. Despite their incipient knowledge, the young people quickly assimilated the information about event-driven PrEP, valuing their autonomy and reducing their daily intake of pills. However, the unpredictability of sexual relations and the difficulty in managing sporadic use were pointed out as impeding factors. Thus, ways of better managing these moments could help to expand use and avoid failures.

Analyzing knowledge without taking into account the practical experience of using the event-driven scheme can limit conclusions, as it is possible that difficulties are overestimated, since they were not contextualized in the health service environment. However, the process of acquiring knowledge, which includes qualified information on prevention, highlights the importance of preventive approaches adapted to the diverse life contexts, information repertoires, preventive practices and specific care needs of young people.
