# Errata

**DOI:** 10.11606/s1518-8787.2025059005729err

**Published:** 2025-06-27

No artigo “Conciliando vantagens e dificuldades: conhecimentos e percepções da PrEP sob demanda entre jovens”, DOI http://doi.org/10.11606/s1518-8787.2020054005729, publicado na Revista de Saúde Pública. 2024;58: Suppl 1:13s, RSP corrige:

**Como citar (página 1):**

Onde se lê:

http://doi.org/10.11606/s1518-8787.2020054005729

Leia-se:

http://doi.org/10.11606/s1518-8787.2024058005729

**Rodapé (páginas 1 a 13):**

http://doi.org/10.11606/s1518-8787.2020054005729

Leia-se:

http://doi.org/10.11606/s1518-8787.2024058005729

