# Supplementary material for: Reconciling advantages and difficulties: knowledge and perceptions of event-driven PrEP among young people
Source: Rev Saude Publica. 2024 Oct 11;58(Suppl 1):13s. doi: 10.11606/s1518-8787.2024058005729 (PMC11573368; doi:10.11606/s1518-8787.2024058005729)
Supplement: Translation

# Translation: Conciliando vantagens e dificuldades: conhecimentos e percepções da PrEP sob demanda entre jovens

**Authors:** Luiz Fabio Alves de Deus, Dulce Ferraz, Lorruan Alves dos Santos, Alexandre Grangeiro, Inês Dourado, Marcia Thereza Couto, Ramiro Fernandez Unsain, Eliana Miura Zucchi

**Affiliations:** I Associação Brasileira Interdisciplinar de AIDS (ABIA). Rio de Janeiro, Brasil Associação Brasileira Interdisciplinar de AIDS Rio de Janeiro Brasil; II Grupo Interdisciplinar de Estudos sobre HIV e Direitos Sexuais e Reprodutivos. Universidade Católica de Santos. Santos, Brasil Grupo Interdisciplinar de Estudos sobre HIV e Direitos Sexuais e Reprodutivos Universidade Católica de Santos Santos Brasil; III Escola de Governo Fiocruz Brasília. Fundação Oswaldo Cruz. Brasília, DF, Brasil Escola de Governo Fiocruz Brasília Fundação Oswaldo Cruz Brasília DF Brasil; IV UMR1296 «Radiations, Défense, Santé, Environnement». Université Lumière Lyon 2. Lyon, France UMR1296 «Radiations, Défense, Santé, Environnement» Université Lumière Lyon 2 Lyon France; V Grupo de estudo e pesquisa em Saúde, Interseccionalidade e Marcadores Sociais da Diferença. Universidade de São Paulo. São Paulo, SP, Brasil Grupo de estudo e pesquisa em Saúde Interseccionalidade e Marcadores Sociais da Diferença Universidade de São Paulo São Paulo SP Brasil; VI Faculdade de Medicina. Universidade de São Paulo. São Paulo, SP, Brasil Faculdade de Medicina Universidade de São Paulo São Paulo SP Brasil; VII Universidade Federal da Bahia. Instituto de Saúde Coletiva. Salvador, BA, Brasil Universidade Federal da Bahia Instituto de Saúde Coletiva Salvador BA Brasil; VIII Universidade de São Paulo. Faculdade de Medicina. Departamento de Medicina Preventiva. São Paulo, SP, Brasil Universidade de São Paulo Faculdade de Medicina Departamento de Medicina Preventiva São Paulo SP Brasil; IX Universidade de São Paulo. Faculdade de Medicina. Programa de Pós-graduação em Medicina Preventiva. São Paulo, SP, Brasil Universidade de São Paulo Faculdade de Medicina Programa de Pós-graduação em Medicina Preventiva São Paulo SP Brasil; X Universidade Católica de Santos. Programa de Pós-Graduação em Saúde Coletiva. Santos, SP, Brasil Universidade Católica de Santos Programa de Pós-Graduação em Saúde Coletiva Santos SP Brasil

**Keywords:** Profilaxia pré-exposição, HIV, Minorias sexuais e de gênero, Adolescente, Pesquisa qualitativa

## Abstract

**OBJETIVO:**

O objetivo deste estudo é analisar o conhecimento sobre o esquema de Profilaxia Pré-Exposição ao HIV sob demanda (PrEP-SD) e a percepção do seu uso potencial entre jovens homens que fazem sexo com homens (HSH), travestis e mulheres trans (TrMT) em acompanhamento na coorte.

**MÉTODOS:**

O estudo qualitativo incluiu 50 entrevistas com participantes das cidades de Salvador e São Paulo, entre 15 e 19 anos, que estavam usando PrEP diária ou outros métodos preventivos. Eles foram abordados por meio de diferentes estratégias de criação de demanda. As entrevistas em profundidade abordaram temas como práticas sexuais, conhecimento sobre PrEP-SD, aceitabilidade e motivações para o uso. Foi realizada análise temática em duas fases usando o software Nvivo versão 12.

**RESULTADOS:**

A maioria dos participantes desconhecia a PrEP-SD e muitos questionaram a sua efetividade e segurança ao receberem informações sobre ela. No entanto, ao conhecer o esquema, muitos jovens perceberam vantagens, como a não obrigatoriedade diária de medicamentos e a possibilidade de uso apenas em momentos de maior risco. Barreiras para o uso da PrEP-SD também foram identificadas pelos participantes, como a imprevisibilidade das relações sexuais e a dificuldade em administrar as dosagens nessa modalidade.

**CONCLUSÕES:**

O conhecimento limitado e as experiências com a PrEP oral diária influenciaram o interesse por ela, o que aponta a necessidade de estratégias informativas que permitam o letramento dos jovens HSH e TrMT na PrEP-SD. Os jovens valorizaram a autonomia e a gestão da prevenção mais alinhada à dinâmica de sua vida sexual propiciada pela nova modalidade, mas enfrentam desafios na gestão da PrEP-SD.

## INTRODUÇÃO

No Brasil, a ampliação do acesso à Profilaxia Pré-Exposição ao HIV (PrEP) é um elemento crucial na resposta à epidemia de HIV entre os grupos mais afetados, como homens que fazem sexo com homens (HSH), travestis e mulheres trans (TrMT), trabalhadores do sexo e parcerias sorodiferentes, sendo necessário dirigir especial atenção à população de jovens homens de 15 a 24 anos, para quem a epidemia tem crescido^1^.

Embora a cobertura da PrEP tenha aumentado entre a população adulta em vários países^2^, os adolescentes e jovens HSH e TrMT enfrentam desafios significativos no acesso, vinculação e retenção à profilaxia, devido a restrições legais, econômicas e sociais que criam barreiras ao longo do *continuum* de cuidado em PrEP^3^^), (^^4^. A dificuldade de acesso e adesão à PrEP entre HSH e TrMT, especialmente menores de 18 anos, decorre de uma gama interrelacionada de fatores individuais, sociais e programáticos, requerendo uma abordagem abrangente e integrada da saúde para o seu enfrentamento^5^^), (^^6^. Evidências também indicam que baixos níveis de percepção de risco de infecção pelo HIV e de conhecimento sobre a PrEP afetam diretamente o interesse pela profilaxia entre indivíduos HSH e TrMT^7^. Elevadas taxas de descontinuidade na utilização da PrEP levantam questões sobre a efetividade desse método em alterar a trajetória da epidemia de HIV entre esses grupos^8^. Estudo conduzido com adolescentes e jovens HSH em Chicago identificou barreiras de acesso aos serviços de saúde, dificuldade em comparecer às consultas de acompanhamento e baixa percepção de risco ao HIV como razões para a descontinuidade da profilaxia^9^. Assim, as barreiras de adesão à PrEP diária enfrentadas por HSH e TrMT, combinadas às especificidades desses grupos, ressaltam a necessidade da oferta de outras modalidades.

Nesse contexto, a modalidade PrEP sob demanda (PrEP-SD) foi recentemente incorporada à política de prevenção do HIV no Brasil, sendo ofertada como alternativa à PrEP diária para grupos específicos, incluindo os HSH e as TrMT, desde que não estejam em uso de hormônios à base de estradiol^10^. Esse esquema de uso da PrEP consiste na ingestão de dois comprimidos de Tenofovir/Entricitabina (TDF/FTC) entre 2 e 24 horas antes da relação sexual de risco, seguidos por mais duas doses, uma 24 e outra 48 horas após a primeira ingestão^11^. Quando utilizada corretamente, a PrEP-SD demonstra eficácia semelhante à PrEP diária^12^ em HSH e TrMT adultas, e é recomendada para indivíduos com práticas sexuais esporádicas e previsíveis^11^^), (^^13^. No entanto, não há estudos específicos sobre a efetividade e segurança do uso da PrEP-SD em HSH e TrMT menores de 18 anos^14^.

Ampliar o conhecimento e a aceitabilidade das diferentes modalidades de PrEP entre jovens e adolescentes HSH e TrMT é importante para promover o direito desses grupos à prevenção. Até o momento, as evidências mostram baixas taxas de conhecimento sobre a modalidade PrEP-SD nessas populações, além de dúvidas sobre sua eficácia e efetividade e desafios relacionados à previsão das relações sexuais e à ingestão dos comprimidos conforme a recomendação^15^. Apesar disso, quando informados sobre os diferentes regimes de PrEP, as taxas de interesse e aceitabilidade da PrEP-SD são elevadas entre os jovens^16^.

Para compreender como adolescentes e jovens respondem à informação sobre a PrEP-SD e sua potencial aceitabilidade, é essencial considerar a construção de sua autonomia, as dinâmicas dos encontros sexuais, as repercussões da orientação sexual e identidade de gênero nos diferentes contextos de vida, especialmente no familiar. Além disso, é importante examinar como essas experiências contribuem para a construção da percepção acerca das diferentes estratégias para prevenção do HIV.

Entendemos por percepção a apropriação e interpretação individual de um fenômeno que influencia comportamentos e práticas em saúde^17^. Assim, esse conhecimento pode ajudar a identificar a intenção de uso e potenciais obstáculos no acesso à profilaxia, contribuindo para o planejamento de ações estratégicas de ampliação de acesso, vinculação e adesão à PrEP nesses grupos. Visando avançar nessa direção, o objetivo deste estudo é analisar o conhecimento sobre o esquema de PrEP sob demanda e a percepção do seu uso potencial entre jovens HSH e TrMT em seguimento na coorte PrEP1519.

## MÉTODOS

Este estudo integra uma investigação qualitativa sobre o acesso a serviços e a vinculação ao cuidado para o HIV e as infecções sexualmente transmissíveis (IST) no contexto de intervenções comunitárias para a oferta de testagem e da PrEP em jovens e adolescentes de São Paulo e Salvador^18^, participantes do estudo PrEP1519.

### O estudo PrEP1519

Iniciado em 2019, o estudo PrEP1519 é a primeira coorte da América Latina com o objetivo de demonstrar a efetividade da PrEP oral diária entre adolescentes HSH e TrMT, com 15 a 19 anos, residentes de três capitais brasileiras: Salvador, São Paulo e Belo Horizonte^19^. O recrutamento ativo da população-alvo para a inscrição na PrEP utilizou diferentes estratégias de criação de demanda. Diversas plataformas virtuais foram utilizadas (Facebook, WhatsApp, Instagram, Twitter, YouTube e Spotify), e educadores de pares interagiram com potenciais participantes em aplicativos de encontros (Grindr, Hornet, Tinder e Badoo). Iniciativas presenciais envolveram a educação entre pares liderada por adolescentes e jovens adultos, que abordaram outros adolescentes em locais de sociabilidade (bares, parques, praias, ruas etc.), além da indicação de profissionais de serviços de saúde especializados em HIV.

Os dados aqui analisados foram produzidos a partir de 50 entrevistas em profundidade com participantes de Salvador e São Paulo, no período de julho de 2019 a setembro de 2020. Buscamos diversificar em relação a: (i) identidades de gênero (homens cis, travestis e mulheres transexuais); (ii) condições sociodemográficas; (iii) experiências de adesão à PrEP; (iv) uso de outros métodos preventivos; e (v) aqueles que ingressaram no estudo por meio de diferentes estratégias de criação de demanda. Os profissionais de saúde e educadores de pares ajudaram a identificar potenciais participantes segundo esses critérios, além de facilitarem o convite para a participação na pesquisa qualitativa.

As entrevistas foram conduzidas com roteiros que abordaram temas como práticas sexuais, uso de métodos de prevenção, aceitabilidade de outras modalidades de PrEP (PrEP-SD, injetável e subcutânea), qualidade de vida e planos futuros. Aos usuários de PrEP foram feitas perguntas sobre adesão ao tratamento e experiências relacionadas ao uso em suas vidas diárias, bem como a interação com os profissionais de saúde. Os participantes que escolheram não utilizar a PrEP no momento da inclusão na coorte foram questionados sobre os motivos de sua recusa e a opção por outros métodos de prevenção. Durante as entrevistas, também foi perguntado aos participantes se conheciam outras modalidades da PrEP além do uso diário, seguido de uma explicação sobre a PrEP-SD.

Entrevistadores treinados e experientes realizaram as entrevistas, que foram conduzidas em sua maioria presencialmente, porém, devido às restrições impostas pela pandemia de covid-19, algumas foram realizadas por videochamada. A duração foi entre 50 e 90 minutos. Todos os participantes deram seu consentimento para participação e gravação das entrevistas, que foram posteriormente transcritas, revisadas e anonimizadas.

A análise dos dados foi conduzida em duas fases distintas e complementares. Na primeira fase, foram desenvolvidos códigos com os temas centrais abordados nas entrevistas, usando uma abordagem dedutiva baseada nos tópicos dos roteiros. Em seguida, as narrativas foram codificadas e estratificadas nos códigos apropriados. Para isso, os participantes foram identificados utilizando uma matriz de extração cruzada, permitindo a análise contextual e individual das narrativas, bem como a comparação entre as percepções acerca da PrEP-SD entre os dois grupos estudados.

A codificação dos dados foi feita com apoio do software NVivo, versão 12. A partir da matriz de codificação, a segunda fase de exploração dos dados foi alinhada ao objetivo do estudo, utilizando os princípios da análise temática^20^. Emergiram três temas estruturantes: (1) o (des)conhecimento sobre o esquema de PrEP-SD; (2) as vantagens e motivações para o uso da PrEP-SD; e (3) as potenciais dificuldades percebidas para um eventual uso do esquema. Ressalta-se que, devido ao caráter inovador da PrEP-SD e às outras possibilidades de prevenção ao HIV, as narrativas se sobrepõem e os temas identificados não são mutuamente excludentes.

Os participantes são citados na seção de resultados por meio de suas falas, identificados por codinome, idade, raça/cor da pele, uso de PrEP ou outros métodos de prevenção, e locais do PrEP1519.

O estudo PrEP1519 foi aprovado pelos Comitês de Ética em Pesquisa da Universidade de São Paulo (protocolo 70798017.3.0000.0065) e da Universidade Federal da Bahia (protocolo 01691718.1.0000.5030). Os participantes maiores de 18 anos assinaram um Termo de Consentimento Livre e Esclarecido (TCLE). Para adolescentes entre 15 e 17 anos, diferentes procedimentos foram adotados: em Salvador, assinaram o Termo de Assentimento Livre e Esclarecido, enquanto os pais ou responsáveis assinaram o TCLE; em São Paulo, mediante autorização judicial, os adolescentes puderam consentir sem a obrigatoriedade de consentimento dos pais ou responsáveis.

## RESULTADOS

Dos 50 entrevistados, 29 eram de Salvador e 21 de São Paulo. Na entrevista, 36 usavam a PrEP diária e 14 faziam uso de outros métodos preventivos. A maioria eram homens cisgêneros (n=42), pretos ou pardos (n=40), entre 18 e 20 anos (n=43), quase metade cursando ensino superior (n=23) e a maioria se identificou como homossexual/gay (n=33) (Tabela 1).

**Tabela 1:** Perfil sociodemográfico dos participantes, uso da profilaxia pré-exposição ao HIV (PrEP) oral diária e conhecimento sobre PrEP sob demanda. Salvador, BA, e São Paulo, SP, Brasil, 2019-2020.

Características	Salvador (N=29)	São Paulo (N=21)
Identidade de gênero		
Homem cisgênero	19 (49%)	20 (51%)
Mulher transgênero	06 (100%)	0 (0%)
Travesti	0 (0%)	1 (100%)
Não binário	04 (100%)	0 (0%)
Faixa etária (anos)		
15-17	3 (43%)	04 (57%)
18-20	26 (60%)	17 (40%)
Raça/corda pele		
Branca	05 (50%)	05 (50%)
Preta	18 (62%)	11 (38%)
Parda	06 (55%)	05 (45%)
Escolaridade		
Ensino médio cursando	06 (55%)	05 (45%)
Ensino médio completo	08 (62%)	05 (38%)
Superior cursando	13 (54%)	11 (46%)
Sem informação	02 (100%)	0 (0%)
Orientação sexual		
Heterossexual	4 (80%)	1 (20%)
Bissexual	6 (60%)	4 (40%)
Gay	18 (55%)	15 (45%)
Pansexual	1 (50%)	1 (50%)
Em uso de PrEP		
Sim	19 (53%)	17 (47%)
Não	10 (71%)	04 (29%)
Conhecimento prévio sobre PrEP sob demanda		
Alguma informação	02 (50%)	02 (50%)
Completamente desconhecida	27 (59%)	19 (41%)

### (Des)conhecimento sobre o regime PrEP-SD

Quase todos os participantes desconheciam a PrEP-SD (46/50). Alguns de São Paulo confundiram a PrEP-SD com a modalidade injetável da PrEP: “Acho que aquela que você aplica de 3 em 3 meses?”(Renato, 19anos, preto, Não PrEP, São Paulo); “Essa sob demanda é aquela que você falou de aplicar no glúteo?” (Girassol, 20 anos, branca, em descontinuidade da PrEP, São Paulo).

Ao receberem informações sobre a PrEP-SD, alguns participantes (seis) ficaram surpresos e apresentaram dúvidas sobre sua efetividade e segurança:

Eu acho que essa, da demanda, meio estranha. [...] Não... o correto seria você já estar prevenido, do que você tomar 2 horas antes do fármaco [...] (Romeu, preto, 19 anos, não usuário de PrEP, Salvador).

Pela informação que eu sei, que protege com sete dias seguidos e não, tipo, dois dias antes e dois dias depois da relação. Eu acho que [a PrEP-SD] não protege (Bernardo, branco, 17 anos, usuário de PrEP, São Paulo).

Dúvidas em relação à interação entre as drogas utilizadas no esquema de PrEP-SD com aquelas utilizadas para a hormonização foram apontadas por uma mulher trans, assim como preocupações com os efeitos colaterais que emergiram tanto nas narrativas de mulheres trans como entre os homens cis: “Eu tenho muito medo de [prejudicar] os rins [...]. Por conta do hormônio e a PrEP também, né” (Vera, preta, 20 anos, não usuária de PrEP, Salvador); “Algum efeito colateral [da PrEP-SD] poderia ser diferente né? O efeito colateral, por ser maior [...]” (Kleber, branco, 20 anos, usuário de PrEP diária, Salvador).

Poucos entrevistados (quatro) tiveram contato prévio com informações sobre a PrEP-SD. A quantidade de comprimidos, o intervalo entre a primeira ingestão e a relação sexual, a quantidade de doses necessárias posteriores à relação sexual e o período de tempo para o uso subsequente eram desconhecidos:

Eu acho que eu já ouvi falar de um só [regime de uso da PrEP]. Eu acho que era dois [comprimidos] sim, um não. Algo do tipo assim, sabe? (Jackson, 17 anos, preto, usuário de PrEP, São Paulo).

Esses dias eu li sobre PrEP três dias antes ou algumas horas antes, eu não lembro. Era uma combinação que era feita alguns momentos antes da transa e depois da transa só, não era algo contínuo. Eu não lembro ao certo a hora ou a ordem, mas eu sei que era algo só no momento da transa e um pouquinho depois da transa (Conrado, 19 anos, branco, não usuário de PrEP, Salvador).

Entre aqueles que afirmaram conhecer a PrEP-SD, apenas um demonstrou conhecimento suficiente para decidir sobre o uso do regime. Para ele, a utilização diária representava uma ameaça à exposição de sua orientação sexual para a família. Aliado à percepção de risco para aquisição do HIV e informações adquiridas em sua rede de sociabilidade, o jovem levou seu interesse no uso eventual da PrEP para a consulta de acompanhamento da PrEP oral diária: “Fiz uma negociação com ele [médico]. Aí depois que ele explicou da PrEP-SD demanda” (Hugo, 20 anos, pardo, usuário de PrEP, São Paulo).

### Vantagens percebidas e motivações para o uso do regime sob demanda

Ao serem apresentados à PrEP-SD, os participantes mencionaram vantagens percebidas no esquema em relação à PrEP diária. A utilização da PrEP apenas quando necessária e a não obrigatoriedade diária da ingestão foram citadas como potenciais vantagens. A compreensão desses aspectos vantajosos partiu da avaliação de que há períodos de pouca frequência nas relações sexuais, permitindo o planejamento. A esporadicidade na exposição ao risco, aliada ao cansaço com a ingestão diária de comprimidos, levou muitos entrevistados a considerarem o regime de PrEP-SD interessante, como observado nas falas:

Gostei. Gostei, sinceramente. Eu acho que eu aderiria. [...] Porque, gente, tipo, ainda mais a situação atual. Porque eu não tô transando muito (Thiago, 18 anos, preto, usuário de PrEP, São Paulo).

Ah, eu acho bem mais legal isso. Por quê? [...] Para mim é muito complicado tomar medicamento todo dia. Eu não curto tomar medicamento todo dia (Guilherme, 20 anos, preto, não usuário de PrEP, São Paulo).

Acho que no caso de situação em que a PREP que eu não queira mais usar, tomar comprimido todos os dias, a PrEP-SD já ajuda, né? (Fernando, 19 anos, pardo, usuário de PrEP, Salvador).

Além disso, o regime foi visto como benéfico para situações de maior risco de aquisição do HIV, como festas de sexo, saunas e dark-rooms. Para alguns participantes que não usavam PrEP, especialmente os em relacionamento monogâmico, a PrEP-SD trazia benefício por ser usada restritamente nos episódios de maior risco:

E eu acho que é muito mais tranquilo, principalmente pra mim, que não gosto de tomar medicamento, e devem ter outras pessoas que também não gostam desse uso contínuo, e seria uma possibilidade se, caso um dia eu queira transar com alguém fora do relacionamento, me preparar antes. (Conrado, branco, 19 anos, Não usuário de PrEP, Salvador).

Eu tomaria. Tipo, eu sei que vou pra uma festa [de sexo] ou então vou ter relacionamento com uma pessoa que eu não conheço, aí eu usaria. Aí eu já sabia, eu usaria logo. Ou então, estou num site de relacionamento, marquei pra sair com a pessoa tal aí, aí vou sair, vou tomar (Danilo, 19 anos, preto, usuário de PrEP, Salvador).

Na PrEP-SD também foram percebidas menores chances de efeitos adversos devido à menor ingestão de comprimidos em comparação à PrEP diária: “Eu acho muito viável, porque você vai ficar tomando todos os dias e nem é todos os dias que você vai ter relação, e aí você acaba tomando, e isso também pode gerar até problemas assim, né?” (Orlando, pardo, 18 anos, não usuário de PrEP, Salvador).

Além disso, um participante notou um benefício coletivo associado à PrEP-SD: a redução do número de pílulas consumidas permitiria um uso mais racional da profilaxia no grupo social, possibilitando que mais pessoas a utilizassem.

“Então não tem necessidade de eu tá tirando tanto comprimido de uma pessoa que poderia estar fazendo o uso, que realmente poderia usar ou precisar, sabe?” (Thiago, 18 anos, preto, usuário de PrEP, São Paulo).

### Potenciais dificuldades para o uso da PrEP sob demanda

Os participantes mencionaram potenciais dificuldades e preocupações com o uso da PrEP-SD. Para alguns (21), a imprevisibilidade das relações sexuais foi destacada como obstáculo, pois os encontros ocorrem de modo repentino, dificultando a tomada da primeira dose até duas horas antes da exposição: “Eu acho a PrEP de uso diário mais eficiente. Porque você nem sempre você sabe que você vai transar” (Theo, branco, 18 anos, usuário de PrEP, São Paulo); “Eu acho que comigo no caso não funcionaria, porque eu nunca prevejo [relações sexuais], nunca marco”. (Paula, branca, 18 anos, usuária de PrEP, Salvador).

A insegurança em relação à proteção em casos de exposição ao risco também foi citada. A quantidade de comprimidos e a orientação para tomar a primeira dose até duas horas antes da relação geraram apreensão sobre a diminuição da eficácia e possíveis efeitos adversos em comparação com a PrEP diária. Alguns (18) demonstraram a preocupação de que a redução do número de pílulas na PrEP-SD, em comparação ao uso diário, resulte em menor proteção contra o HIV:

Ah, eu acho que talvez funcionaria. Mas, pela informação que eu tenho, não faria, não. Pela informação que eu sei, que protege com sete dias seguidos, e não, tipo, dois dias antes e dois dias depois da relação. Eu acho que não protege (Bernardo, 17 anos, branco, usuário de PrEP, São Paulo).

Tem vezes que você acha que não vai acontecer. Por exemplo, a última vez que eu tive relação sexual eu não achei que ia ter. Então tem coisas que você fica desprevenido, se você não tiver com essa regularidade que o comprimido te possibilita (Walter, 19 anos, preto, usuário de PrEP, Salvador).

Eu acho que essa da demanda [é] meio estranha [...]. Não, [...] o correto seria você já estar prevenido, do que você tomar 2 horas antes do fármaco e você achar que vai ter um efeito assim (Romeu, preto, 19 anos, não usuário de PrEP, Salvador).

Além disso, alguns participantes (oito) se sentiram incapazes de seguir a recomendação de uso da PrEP-SD:

Pesado. Pesadíssimo isso aí. Por quê? Ah, não sei, [por]que corre o risco do esquecimento humano, né? (Luiz, 20 anos, preto, usuário de PrEP, São Paulo).

A PrEP-SD eu não usaria por questão de, tipo assim, eu tenho dificuldade de lembrar das coisas, tanto que inicialmente me adaptar a tomar o medicamento foi complicado, e ainda assim eu tenho que anotar quando eu tomo o medicamento (Roberto, preto, 16 anos, usuário de PrEP diária, Salvador).

Por fim, embora menos mencionada, houve preocupação em compatibilizar o uso da PrEP-SD em contextos de alta atividade sexual, tanto com parceiro fixo quanto casual:

Veja bem, se for [...], são quatro. Se você tiver duas relações na semana, aí já vão ser oito, oito comprimidos, então é [...] (Carlos, 19 anos, preto, usuário de PrEP, Salvador).

Pra mim, por demanda não ia ser necessário, porque eu namoro, tenho um relacionamento permanentemente, sabe, corriqueira, então “por demanda” se tornaria diariamente, entendeu?. (Luciano, 18 anos, pardo, usuário de PrEP, Salvador).

## DISCUSSÃO

O conhecimento sobre a PrEP-SD não fazia parte do repertório sobre prevenção ao HIV dos jovens HSH e TrTM. Os poucos participantes com alguma informação prévia sobre a PrEP-SD afirmaram fazer uso da profilaxia quando têm relações sexuais, mas sem um completo conhecimento de como utilizar adequadamente o esquema. Sendo um conhecimento ainda incipiente, a expressão “sob demanda” foi confundida com a PrEP injetável entre alguns, assim como informações sobre efetividade e formas de uso foram confundidas com a de uso diário. Investigações com diferentes abordagens metodológicas têm demonstrado que o conhecimento sobre a PrEP-SD é baixo mesmo entre usuários de PrEP diária^21^^), (^^22^^), (^^23^. E, entre aqueles que informaram ter algum conhecimento sobre a modalidade, quando incentivados a apresentar a informação sobre as dosagens e o esquema de uso pré e pós relação sexual, poucos o reportaram corretamente, como demonstrado também em outros estudos^21^^), (^^22^^), (^^23^^), (^^24^. Esse, inclusive, tem sido um dos principais problemas que levam ao uso incorreto do esquema^25^.

Adicionalmente, entre as TrMT, houve preocupação de que a interação entre os fármacos presentes na PrEP-SD e substâncias utilizadas para a hormonização resultem em danos renais, preocupação similar identificada em estudo estadunidense com mesmo grupo^26^. No entanto, esse grupo não destacou a preocupação com a possibilidade de interação entre o uso do hormônio e a eficácia do esquema, o que poderia contraindicar a utilização da PrEP-SD para essas pessoas^27^. Esses resultados reiteram outros achados que realçam como HSH e TrMT são negativamente afetados no que tange ao acesso à informação sobre métodos preventivos baseados no uso de antirretrovirais^23^. Em um contexto de elevada ocorrência de HIV entre HSH e TrMT, como apresentado em estudos realizados no Brasil^28^, o desconhecimento sobre a PrEP-SD revela a necessidade de desenvolvimento e inclusão da informação sobre os diferentes regimes de PrEP nas ações de prevenção combinada ao HIV que considere especificidades e necessidades dos subgrupos populacionais mais vulneráveis. Isso pode levar a um aumento da cobertura de PrEP em grupos mais suscetíveis pela epidemia, atingindo pessoas com diferentes comportamentos sexuais e diferentes necessidades de prevenção.

Corroborando esses aspectos, mesmo demonstrando conhecimento incipiente, os jovens HSH e TrMT se mostraram capazes de assimilar rapidamente as informações sobre o regime sob demanda e, ao avaliar as características de suas relações sexuais, mediadas ou não pela experiência de uso da PrEP diária, identificaram vantagens e intenção de uso da PrEP-SD. De maneira geral, os benefícios reconhecidos estavam atrelados à percepção de maior autonomia em relação à tomada de decisão sobre o regime de PrEP a ser utilizado. Na medida em que diferentes regimes de PrEP estão disponíveis e, considerando que no campo da prevenção ao HIV o melhor regime é aquele que considera os valores, as diferentes expectativas, necessidades e realidades dos indivíduos, a tomada de decisão centrada no usuário se apresenta como uma estratégia importante para o avanço e disseminação do cuidado no contexto da prevenção do HIV^29^. Ao considerar a modalidade PrEP-SD vantajosa, os participantes levaram em conta a própria capacidade de administrar uma rede complexa de elementos que se apresentam como fundamentais para a tomada de decisão, tais como a frequência e sazonalidade da atividade sexual e a capacidade de prever e planejar os encontros sexuais.

Nesse sentido, abordagens que permitam um processo dialógico entre profissionais de saúde e usuários no processo de tomada de decisão sobre o regime de PrEP parecem ter melhores chances de êxito^29^^), (^^30^. Investigações realizadas majoritariamente com HSH têm identificado que aspectos relacionados às dinâmicas dos encontros sexuais, tais como a capacidade de planeja-los, a irregularidade ou diminuição em sua frequência^25^^), (^^30^^), (^^31^^), (^^32^^), (^^33^^), (^^34^^), (^^35^^), (^^36^^), (^^37^^), (^^38^, a baixa percepção de risco para a aquisição do HIV^25^^), (^^36^^), (^^39^, além de preocupação com o uso excessivo de medicação^21^^), (^^29^^), (^^30^^), (^^32^^), (^^33^ e receio ou experiência com efeitos colaterais^23^^), (^^36^^), (^^39^, exercem influência na escolha inicial pela PrEP-SD ou mudança da modalidade de uso diário para a sob demanda. Além disso, podem influenciar num adequado processo de gestão do uso da PrEP, permitindo intercalar o uso diário e sob demanda de acordo com o perfil das relações sexuais em cada momento.

Além do reconhecimento da maior conveniência, flexibilidade e praticidade do regime PrEP-SD, contrapostas à percepção de que a PrEP diária, em determinadas circunstâncias, pode não ser a melhor opção por conta da ingestão diária de comprimidos, também identificados em outras pesquisas qualitativas^13^^), (^^32^, a PrEP-SD emergiu como alternativa interessante para aqueles indivíduos em relacionamento monogâmico, mas que eventualmente se envolvem em relações sexuais que escapam do acordo de exclusividade. É importante destacar a preocupação com as infecções por HIV que ocorrem em contextos de relacionamentos monogâmicos, pois essas situações também têm sido observadas em casos de usuários de PrEP, que interrompem a profilaxia ao iniciarem relacionamentos monogâmicos por não se perceberem em risco^40^. Diante disso, é crucial que esse aspecto seja abordado durante o aconselhamento, a fim de permitir uma tomada de decisão adequada sobre as estratégias preventivas mais seguras para essas situações.

De forma semelhante a outras investigações^41^, os resultados deste estudo mostram que a PrEP-SD também foi percebida como tendo potencial benefício coletivo, por resultar em uso mais racionalizado e capaz de ampliar a oferta para mais pessoas, por meio de melhor utilização dos comprimidos disponíveis no sistema de saúde. Indiretamente, essa percepção de benefício coletivo também parece indicar a percepção dos jovens sobre a necessidade de adaptações em contextos de limitação dos insumos de prevenção.

Em oposição às vantagens percebidas da PrEP-SD, foram tecidas considerações sobre potenciais dificuldades para o uso da modalidade. Em um primeiro plano, um eventual uso da PrEP-SD aparece como dificultado pela imprevisibilidade dos encontros sexuais, barreira que parece comum quando se trata da PrEP-SD^33^^), (^^35^^), (^^37^. A dificuldade em prever e planejar as relações sexuais pode estar mediada pela imediaticidade dos encontros sexuais. Aqui cabe destacar que o aumento do uso de aplicativos para encontro de parceiros sexuais tem sido apontado como uma vantagem para o planejamento do uso da PrEP-SD, na medida em que marca o momento para o início do uso da modalidade. Por outro lado, é importante considerar que muitos adolescentes e jovens adultos residem com pais e/ou outros familiares que desconhecem seus desejos e práticas sexuais^42^. Nesse contexto, a limitação da autonomia e independência, inclusive financeira, podem contribuir com a percepção de que certas oportunidades de vivência da sexualidade são únicas, o que pode criar uma barreira para o uso das dosagens iniciais da PrEP-SD conforme a recomendação. A capacidade de administrar o uso de todos os comprimidos do esquema emergiu como dificultador do regime PrEP-SD, percebido como complexo e propício ao esquecimento, consonantes com achados de outras investigações^33^^), (^^35^^), (^^38^. Dessa forma, uma especificidade importante de jovens e adolescentes consiste em reconhecer a autonomia para o cuidado como um processo de aprendizagem que requer lidar com a complexidade de decisões e da interação com os contextos das relações familiares e sociais, bem como da organização da rotina de cuidado integradas à dinâmica dos encontros sexuais para administrar o uso eventual da PrEP.

Além disso, é notória a necessidade de que as estratégias de divulgação e promoção da PrEP-SD tenham como um dos focos a desconstrução de crenças equivocadas sobre a sua eficácia e efetividade, como as identificadas neste e em outros estudos^23^^), (^^32^. Estudo realizado com homens gays e bissexuais australianos identificou que a crença da eficácia estava associada ao conhecimento prévio do método^24^. Entre os nossos participantes, as dúvidas que emergiram sobre a eficácia da PrEP-SD parecem estar atreladas à novidade da informação sobre o método e à experiência de uso e maior exposição a informação sobre a PrEP diária, como a necessidade de uso do medicamento por sete dias para se obter a concentração necessária no organismo para garantir uma proteção adequada. Nesse sentido, parece adequada a recomendação para disponibilizar a modalidade diária e sob demanda em um único esquema oral, unificando os procedimentos de início (dose de ataque com dois comprimidos), manutenção (um comprimido por dia de relação) e finalização (dois comprimidos após a última potencial exposição)^10^.

## CONCLUSÕES

O baixo conhecimento e as percepções baseadas na experiência com a PrEP diária influenciaram o interesse e a decisão sobre a PrEP-SD, o que ressalta a necessidade de aprimorar as estratégias de informação e oferta desse esquema nas estratégias de prevenção combinada para jovens HSH e TrMT. Apesar do conhecimento incipiente, os jovens assimilaram rapidamente as informações da PrEP-SD, valorizando sua autonomia e redução na ingestão diária de comprimidos. Porém a imprevisibilidade das relações sexuais e a dificuldade em gerenciar o uso esporádico foram apontados como impeditivos, assim, formas de melhor gerir esses momentos podem auxiliar na expansão do uso, assim como evitar falhas.

Analisar o conhecimento sem considerar a experiência prática do uso do regime sob demanda pode limitar as conclusões, pois é possível que as dificuldades sejam superestimadas, uma vez que não foram contextualizadas no ambiente de atendimento do serviço de saúde. No entanto, o processo de adquirir conhecimento, que inclui a informação qualificada sobre prevenção, destaca a importância de abordagens preventivas adaptadas aos diversos contextos de vida, repertórios de informações, práticas preventivas e necessidades de cuidado específicas dos jovens.
