# Supplementary material for: Errata
Source: Rev Saude Publica. 2025 Jun 27;59:e13serr. [Article in Portuguese] doi: 10.11606/s1518-8787.2025059005729err (PMC12207893; doi:10.11606/s1518-8787.2025059005729err)
Supplement: Related texts

# Correction: Erratum

In the article “Reconciling advantages and difficulties: knowledge and perceptions of event-driven PrEP among young people”, DOI http://doi.org/10.11606/s1518-8787.2020054005729, published on the Revista de Saúde Pública. 2024;58: Suppl 1:13s, RSP corrects:

**How to cite (page 1):**

Where it reads:

http://doi.org/10.11606/s1518-8787.2020054005729

It should read:

http://doi.org/10.11606/s1518-8787.2024058005729

**Footnote (pages 1 to 13):**

Where it reads:

http://doi.org/10.11606/s1518-8787.2020054005729

It should read:

http://doi.org/10.11606/s1518-8787.2024058005729
